# Sexual risk behaviours of high school female learners in Mbonge subdivision of rural Cameroon

**DOI:** 10.11604/pamj.2015.20.49.2938

**Published:** 2015-01-19

**Authors:** Elvis Enowbeyang Tarkang

**Affiliations:** 1 HIV/AIDS Prevention Research Network, Cameroon (HIVPREC) Kumba, South West Region, Cameroon

**Keywords:** Sexual risk behaviours, high school female learners, HIV/AIDS, Mbonge subdivision, rural Cameroon

## Abstract

**Introduction:**

Since female learners in high schools in Cameroon fall within the age group hardest hit by HIV/AIDS, it is assumed that these learners might be exposed to sexual risk behaviours. However, little has been explored on the sexual risk behaviours of high school female learners in Cameroon. This study aimed at examining the sexual risk behaviours of high school female learners in Mbonge subdivision of rural Cameroon.

**Methods:**

A cross sectional design was adopted, using a self-administered questionnaire for data collection. Respondents were selected through disproportional stratified simple random sampling resulting in 210 female grade 10 to grade 12 learners from three participating high schools in Mbonge subdivision, Cameroon. Descriptive and inferential statistics were calculated using SPSS version 20 software program.

**Results:**

Majority of the respondents, 54.0% reported being sexually active, of whom only 39.8% used condoms during first sex; 49.5% used condoms during last sex and 29.6% used condoms consistently. Up to 32% of the sexually active respondents had multiple sexual partners in the past one year before the study, while 9.3% had multiple sexual partners during the study period. The mean age of first sex was 15.6 years. Lack of parental control, religion, academic profile, poverty, place of residence and perception of risk of HIV infection were the main factors significantly associated with sexual risk behaviours.

**Conclusion:**

The findings indicate that sexual risk behaviours exist among high school female learners in Mbonge, Cameroon. There is need for campaigns and interventions to bring about sexual behaviour change.

## Introduction

HIV/AIDS remains a global challenge. Of the 34 million people living with HIV/AIDS worldwide in 2010, almost 68% reside in sub-Saharan Africa (SSA), a region with only 12% of the world's population [1]. Young people are the most threatened globally. In SSA, young people aged 15-24 accounted for half of all new HIV infections in 2009 [1, 2]. Therefore monitoring the sexual behaviours of this vulnerable age group is necessary in order to control the HIV/AIDS pandemic. Sexual risk behaviours include engaging in unprotected vaginal intercourse, early sexual debut, multiple sexual partners and coerced or forced sex [3–5]. These risky sexual practices are influenced by many factors including the lack of accurate information on the modes of transmission of HIV/AIDS, economic conditions, gender inequalities, living place, religion, perception of risk of HIV infection, age and academic profile [6–11]. Young people are particularly vulnerable to HIV infection because of risky sexual behaviours and substance abuse. These behaviours are convoluted by lack of access to accurate and personalised HIV information and prevention services and many other socio-economic reasons, living away from parents and free from parental control, peer pressure and lack of youth friendly recreational facilities [12]. Despite a wealth of research on youth, little research has been done on the sexual risk behaviours of high school learners in Cameroon. Thus this study aims to examine the sexual risk behaviours of high school female learners in Mbonge rural area of Cameroon, for possible interventions.

## Methods

The design used to examine the sexual risk behaviours of high school female learners in Mbonge rural area of Cameroon, was descriptive and correlational, collecting data through self-administered questionnaires. The questionnaire was pretested to clarify instructions, relevancy, usability and completion time, to refine and introduce modifications where necessary and to ascertain reliability and validity [13]. During the pre-test, 10 students, who did not participate in the actual study, completed the questionnaires. They required no assistance, understood the questions and needed approximately 30 minutes to complete the questionnaires. The reliability of the research instrument used for the study was tested by pre-testing the questionnaires. The following types of validity were also established: face validity, content validity, construct validity and criterion-related validity. This was ensured by constructing items to represent the different components of the study, based on literature review. The questionnaires were distributed 210 female learners in three high schools in Mbonge rural area of Cameroon during normal class periods with the permission of the principals and the co-operation of the teachers concerned. One research assistant was available to assist the learners and to answer questions while they completed the questionnaires during a classroom period. To obtain the sample, the researcher used the school attendance registers of the learners as a sampling frame. Data collection took place during the first term of 2012.

Permission to conduct this study was granted by the HIV/AIDS Prevention Research Network, Cameroon (HIVPREC), a Non-governmental Organisation (NGO) for the prevention of HIV/AIDS through formalized education, working in the South West region of Cameroon, and the principals of the three participating high schools. Participation was voluntary and informed written consent was obtained from each learner and her parent/guardian prior to data collection. A questionnaire was handed to a learner when she produced the signed consent form from a parent/guardian and from herself. Anonymously completed questionnaires were kept in a separate container from the signed informed consent forms in order to maintain anonymity. Anonymity was also maintained by reporting the findings of the three schools combined and by not providing comparisons among the three schools. Confidentiality was maintained because only the researcher had access to the completed questionnaires, which were locked up. Subsequent to the acceptance of the research report, these would be destroyed. Data were collected in three sections: socio demographic variables, knowledge of prevention of HIV/AIDS and knowledge of sexual risk behaviours, and sexual behaviours.

**Data analysis**: data were analysed using SPSS version 20. Data were summarised by means of descriptive statistics including the frequency table. More advanced statistics included the chi square test at the 0.05 significant level.

## Results

The socio-demographic characteristics of the respondents are shown in Table 1. A total of 210 female learners responded to the questionnaires. The mean age (SD) of the respondents was 18.1 years (1.8) (data not shown). All were high school female learners, of whom 194 (93.3%) were single. Most, 150 (72.5%) passed their exams on merit, thus indicating a high academic profile. Majority, 195 (92.8%) were Christians, of whom 178 (84.7%) were orthodox (non-Pentecostal) Christians. Most, 163 (79.2%) were resident in the cities and townships, while 57 (27.4%) were living in rented places during school period. Regarding parents’ average monthly incomes, 55 (31.3%) and 86 (46.5%) stated that their fathers’ and mothers’ monthly incomes were less than 100 000XAF (US$200.00) respectively. Table 2 presents knowledge regarding prevention of HIV/AIDS, knowledge regarding sexual risk behaviours and perception of HIV infection risk among the learners. Most learners, 145 (72.5%) believed that correct and consistent use of condoms could prevent HIV infection; most, 186 (91.6%) believed that sexual abstinence could prevent HIV infection, and 168 (83.1%) believed that being faithful to one uninfected sexual partner could prevent HIV infection. With regard to knowledge of sexual risk behaviours, majority, 124 (70.9%) believed that early sexual debut is a sexual risk behaviour. In the same vein, 140 (79.5%) believed that unprotected sexual intercourse is a sexual risk behaviour, and 141 (76.2%) believed that having multiple sexual partners is a sexual risk behaviour. Majority of the learners, 119 (66.5%) also believed that coerced or forced sex is a sexual risk behaviour.

**Table 1:** Socio-demographic characteristics of high school female learners in Mbonge, Cameroon

Characteristics	Frequency	Percentage
**Age Group (210)**		
11-15	16	7.6
16-24	194	92.4
**Marital Status (208)**		
Single	194	93.3
Married	3	1.4
Divorced	2	1.0
Cohabiting	4	1.9
Others	5	2.4
**House of residence (203)**		
Duplex	10	4.9
5 rooms or more	97	47.8
4 rooms or less	79	38.9
Shack	10	4.9
Others	7	3.4
**Academic profile (207)**		
Passed	150	72.5
Promoted on trial	11	5.3
Repeated	46	22.2
**Religious Affiliation (210)**		
Catholic	74	35.2
Jehovah's witness	4	1.9
Presbyterian	100	47.6
Pentecostal	17	8.1
Muslim	4	1.9
Others	11	5.2
**Present residential area (206)**		
City	65	31.6
Suburb	4	1.9
Rural area	37	18.0
Informal setting	2	1.0
Township	98	47.6
**Living place during school period (208)**		
Boarding	6	2.9
Rented place	57	27.4
Parent's house	96	46.2
Guardian's house	48	23.1
Others	1	0.5
**Father's monthly income (176)**		
300 000XAF and above	50	28.4
200 000-300 000XAF	35	19.9
100 000-200 000XAF	36	20.5
100 000XAF or less	55	31.3
**Mother's monthly income (185)**		
300 000XAF and above	26	14.1
200 000-300 000XAF	24	13.0
100 000-200 000XAF	49	26.5
100 000XAF or less	86	46.5

**Table 2:** Knowledge of prevention of HIV/AIDS, Knowledge of sexual risk behaviours and perception of risk of contracting HIV/AIDS of high school female learners in Mbonge, Cameroon

Variables	Frequency	Percentage
**Correct and consistent use of condoms can prevent HIV/AIDS(200)**		
Agree	145	72.5
Disagree	55	27.5
**Sexual abstinence can prevent HIV/AIDS (203)**		
Agree	186	91.6
Disagree	17	8.4
**Being faithful to one sexual partner can prevent HIV/AIDS (202)**		
Agree	168	83.1
Disagree	34	16.9
**Early sexual debut is a sexual risk behaviour (175)**		
Agree	124	70.9
Disagree	51	29.1
**Unprotected sexual intercourse is a sexual risk behaviour (176)**		
Agree	140	79.5
Disagree	36	20.5
**Having multiple sexual partner is a sexual risk behaviour (185)**		
Agree	141	76.2
Disagree	44	23.8
**Coerced or forced sex is a sexual risk behaviour (179)**		
Agree	119	66.5
Disagree	60	33.5
**Perception of risk of contracting HIV/AIDS (203)**		
Not at risk	89	43.8
Small risk	18	8.9
Moderate risk	16	7.9
High risk	80	39.4

Regarding the perception of risk of HIV infection, less than half of the learners, 80 (39.4%) indicated that they were at high risk of HIV infection. Table 3 explicates the sexual behaviours of the learners. The majority, 108 (54.0%) reported having experienced sex. Learners who lived in rented places, 36 (66.7%) were more likely to have ever had sex than those who lived in their parents’ or guardians’ houses, 59 (43.1%) (p = 0.034), while learners with low academic profiles, 41 (74.5%) were more likely to have experienced sex than those with high profiles, 58 (40.6%) (p = 0.000). The mean age (SD) at first sex was 15.6 years (3.1). By age 16 years, the majority, 58 (59.2%) had already experienced sex. Among learners who ever had sexual intercourse, 23 (20.4%) were forced into sex during their first sexual encounters, while 17 (15.0%) were influenced by their friends. Learners who lived in rented places, 19 (41.3%) were more likely to have experienced sexual coercion than those who lived in their parents’ or guardians’ houses, 32 (40.5%) (p = 0.007). Learners with high academic profiles, 18 (26.9%), were more likely to have been forced by their partners during first sex, than those with low profiles, 4 (9.8%) (p = 0.000). Learners who perceived themselves at high risk of contracting HIV/AIDS, 28 (47.5%), were more likely to have been forced by their partners into first sex than those who perceived themselves not at risk, 12 (26.1%) (p = 0.016). Learners resident in the suburbs and rural settings, 7 (24.1%) were more likely to have been forced by their partners into first sex than those resident in the townships and cities, 17 (21.8%) (p = 0.040). Christians, 22 (20.8%) were more likely to have been forced into first sex than Muslims, 0 (0.0%); while among the Christians, non-Pentecostals, 21 (21.4%) were more likely to have been forced into first sex than Pentecostals, 1 (12.5%) (p = 0.002). Among the Christians, Pentecostals, 7 (87.5%) were more likely to have experienced sex by age 16 years than non-Pentecostals, 51 (58.6%) (p = 0.004). Male dominance and the subservient position of women and children in some African societies contribute to coerced sex.

**Table 3:** Sexual behaviours of high school female learners in Mbonge, Cameroon

Variables	Frequency	Percentage
**Ever had sexual intercourse with a male partner (200)**		
Yes	108	54.0
No	92	46.0
**Age at which first sexual intercourse occurred (98)**		
16 years or less	58	59.2
More than 16 years	40	40.8
**How first sexual intercourse happened (113)**		
Was forced	23	20.4
Was planned	27	23.9
Was influenced by friends	17	15.0
It just happened	35	31.0
Curiosity	7	6.2
Others	4	3.5
**Number of sexual partners in the past one year (97)**		
None	12	12.4
One	54	55.6
Two or more	31	32.0
**Number of concurrent sexual partners at present (97)**		
None	20	20.6
One	68	70.1
Two or more	9	9.3
**Condom use during first sex (103)**		
Yes	41	39.8
No	62	60.2
**Condom use during last sex (105)**		
Yes	52	49.5
No	53	50.5
**Ever used condoms (97)**		
Yes	73	75.3
No	24	24.7
**Regularity of condom use (108)**		
Always	32	29.6
Most of the time	33	30.6
Seldom	14	13.0
Never	29	26.8

Among learners who had experienced sex, 31 (32.0%) had multiple sexual partners in the past one year before the study period, while 9 (9.3%) had multiple concurrent sexual partners during the study period. Learners who perceived themselves to be at high risk of HIV infection, 13 (16.7%) were more likely to have had multiple sexual partners in the past one year than those who perceived themselves not at risk, 11 (12.6%) (p = 0.012). Learners who lived in rented places, 17 (32.1%) were more likely to have had multiple sexual partners in the past one year than those who lived in their parents’ or guardians’ houses, 16 (11.4%) (p = 0.001). Learners with low academic profiles, 14 (26.0%) were more likely to have had multiple partners in the past one year than those high profiles, 19 (13.0%) (p = 0.002). In the same vein, learners with low academic profiles 4 (7.4%) were more likely to have had multiple concurrent sexual partners during the period of this study, than those with high profiles, 8 (5.4%) (p = 0.000). Learners resident in the suburbs and rural settings, 15 (36.6%) were more likely to have had multiple sexual partners in the past one year than those resident in the cities and townships, 18 (11.5%) (p = 0.006). The proportion of condom use was quite low. Among learners who reported to have ever had sexual intercourse, 73 (75.3%) had used a condom at least once. Less than half, 41 (39.8%) had used condoms during their first sexual encounters. Learners who perceived themselves at high risk of HIV infection, 21 (42.9%) were more likely to have used condoms during first sex than those who perceived themselves not at risk, 11 (30.6%) (p = 0.035). Learners who lived in their parents’ or guardians’ houses, 28 (50.0%), were more likely to have used condoms during first sex than those who lived in rented places, 12 (28.6%) (p = 0.000). Learners with high academic profiles, 26 (40.6%) were more likely to have used condoms during first sex than those with low academic profiles, 15 (39.5%) (p = 0.025). Learners resident in the townships and cities, 36 (48.6%) were more likely to have used condoms during first sex than those resident in the suburbs and rural settings, 5 (13.5%) (p = 0.009).

Condom use in most recent sexual encounters was also low, 52 (49.5%), although higher than use during first sexual encounters. Learners who lived in rented places, 21 (50.0%) were more likely to have used condoms during last sex than those who lived in their parents’ or guardians’ houses, 28 (48.3%) (p = 0.003). Learners who passed their examinations, 35 (53.8%) were more likely to have used condoms during last sex than those with low academic profiles, 17 (43.6%) (p = 0.001). Consistent condom use was low, 32 (29.6%). Learners who perceived themselves not to be at risk of HIV infection, 15 (40.5%) were more likely to use condoms consistently than those who perceived themselves to be at high risk, 16 (30.8%) (p = 0.001). Learners who lived in rented places, 14 (33.3%) were more likely to use condoms consistently during sex, than those who lived in their parents’ or guardians’ houses, 17 (27.9%) (p = 0.040). Learners who passed their examinations, 22 (32.8%) were more likely to have used condoms consistently than those with low academic profiles, 10 (25.0%) (p = 0.001).

## Discussion

Majority of the respondents were aware of the measures to prevent HIV/AIDS: correct and consistent condom use (72.5%), abstaining from sex (91.6%) and being faithful to one sexual partner (83.1%). These percentages are higher than those obtained by Rwenge in Bamenda in 2000 [14], and also higher than those obtained in Rwanda [15]. Knowledge of sexual risk behaviours was also high: early sexual debut (70.9%), unprotected sex (79.5%), multiple sexual partners (76.2%) and coerced sex (66.5%). However, less than half (39.4%) of the respondents felt they were at high risk of HIV infection. This percentage is higher than those in Tiko, Cameroon (18.8%) [16] and in Rwanda (3.2%) [15]. This disparity might be because of the interventions that have been given at different levels in Cameroon. Nearly all (93.3%) the respondents were unmarried, thus restricting the study to single young females. One hundred and eight (54.0%) respondents reported ever having had sex. This is similar to that obtained in Tiko, Cameroon (47.3%) [16], but higher than those obtained in Rwanda (33.0%) [15] and in Ethiopia (28%) [17]. This disparity could be due to the cultural differences in relation to sexual activity between these two countries and Cameroon.

The study showed that sexual risk behaviours is common among the respondents. The respondents reported a very young age of sexual debut (15.6 years). This is similar to those obtained in Bamenda, Cameroon (15.8%) [18], in Tiko, Cameroon (16.9%) [16], in Rwanda (15 years) [15] and in Ethiopia (17.5%) [17]. The early age of sexual debut might indicate that the issue of early sexual initiation is a common problem in Africa. Twenty three (20.4%) respondents said their first sex was forced, which is less than that obtained in Bamenda (30.0%) [14], but similar to that obtained in Ethiopia (12.2%) [17]. The decrease in incidence of forced sex in Cameroon might be due to the behavioural interventions that have taken place at different levels. Among the respondents who had experienced sex, 32.0% had multiple sexual partners in the one year before the study period, which is similar to that obtained in Tiko, Cameroon (42.8%), [16], Rwanda (33.0%) [15] and Ethiopia (33.5%) [17]; while 9.3% had multiple sexual partners at the time of the study which is lower than results obtained in Bamenda Cameroon (27.0%) [18] and Ethiopia (24.5%) [17]. This indicates that despite interventions, the behaviour of learners regarding multiple sexual partners remains high in Africa.

The overall prevalence of ever use condoms among the sexually active respondents was 75.3%, which is similar to that obtained in Ethiopia (64.1%) [17]. Only 39.8% of the sexually active respondents used condoms at first sex, which is higher than results obtained in Rwanda (8.3%) [15]. This disparity might be due to cultural background differences between Cameroon and Rwanda. Fifty two (49.5%) sexually active respondents used condoms during the last sexual intercourse, which is higher than results obtained by Rwenge in Bamenda (25%) [18] and that obtained in Rwanda (13.2%) [15]. This might be due to the behavioural interventions that have taken place at different levels in Cameroon. The disparity between Cameroon and Rwanda could be due to cultural background differences between the two countries. However, consistent condom use as reported by the sexually active respondents was low (29.6%), but higher than that obtained in Ethiopia (20.4%) [17]. This could also be due to cultural background differences between Cameroon and Ethiopia in relation to sexual activity. The following socio-demographic factors were identified in this study as having significant influence on sexual risk behaviours (early sexual debut, multiple sexual partners, low condom use and coerced or forced sex) among high school female learners in Mbonge subdivision of rural Cameroon: low perception of risk of HIV infection, low academic profile, being a Christian (Pentecostal), living alone during school, living in a suburb/rural setting and poverty [6–8].

Poor female learners may be pushed into sexual risk behaviours to ensure survival, receive material goods to relieve poverty; and this behaviour is likely to be associated with increased HIV risk. Low socio-economic status appears to be associated with learners having experienced coerced sex [9]. Living alone, away from parents is an important factor associated with adolescents’ behaviours. Youth who live with both parents appear to be less likely to engage in sexual risk behaviours than those living alone. Living in a family with either parents or guardians present implies the availability of support, supervision and behavioural control in the lives of adolescents [19]. Learners living in the cities and townships have easy access to formalised information regarding HIV/AIDS and preventive measures than their counterparts living in the suburbs and rural settings. This gives them the opportunity to engage in safe sexual practices. Concerning religion, it is significant that a high religiosity made adolescents less likely to engage in premarital sex; teenagers, who attended religious services regularly, delayed the timing of their first sexual encounters; adolescents who are highly religious are less likely to use condoms during sexual intercourse [20–22]. A high level of academic engagement has an influence on the age of sexual initiation and makes health education messages more meaningful [23, 24]. Adolescents with high academic aspirations are more likely not to jeopardise their academic careers by unwanted pregnancies and STDs, including HIV/AIDS, by abstaining from sex, by being faithful to one sexual partner, or by using condoms when engaging in sexual intercourse with multiple partners.

Information on sexual risk behaviours is important for designing and monitoring intervention programmes to curb the prevalence of HIV/AIDS especially among female youth. The respondents in this study were well informed about HIV/AIDS, methods of prevention and sexual risk behaviours. Despite such knowledge, they continue to engage in sexual risk behaviours that could expose them to the risk of HIV infection. Majority of the female learners were sexually active, the majority were engaged in early sexual debut and many also had sex with multiple partners. Condom use was very low and those that used them did not use them consistently. Concerning perception of risk of HIV infection, only 39.4% of respondents perceived themselves at high risk of HIV infection. This low risk perception, coupled with low level of condom use, early sexual debut and multiple sexual partners strongly suggest that HIV prevention campaigns among females in Mbonge subdivision of rural Cameroon are not being translated into safe sexual practices, and therefore calls for reinforcement efforts.

This study was conducted in predominantly Christian area of Cameroon. Different results may be obtained if the study is conducted on predominantly Muslim learners. The homogenous nature of the sample limits generalization of the findings. Only questionnaires were used to collect data. More in-depth data might have been obtained if individual interviews could have been conducted. However the learners’ responses to questionnaire items were accepted as their sexual behaviours because the truthfulness of their answers could not be ascertained.

## Conclusion

The findings of this study suggest that female learners in Mbonge subdivision, Cameroon have needs in terms of HIV/AIDS education and prevention. All sexuality education programmes and HIV/AIDS education should commence before the age of 15 years. The “ABC” prevention programmed should be emphasised, especially consistent condom use, correct use of condoms and condom negotiation skills. Abstinence should be emphasised, and faithfulness as a value should be reinstated in sexual relationships. HIV prevention programmes targeted at female learners in Mbonge subdivision should also aim at increasing the perception of HIV infection risk among them. The prevalence of HIV among Cameroon female youths is unlikely to decrease until these youths practice safe sex. Health education messages apparently succeeded in imparting knowledge but not in changing youths’ sexual behaviours.

## References

[1] UNAIDS (2010). Report on the global AIDS epidemic.

[2] UNAIDS (2011). World AIDS day report: How to get zero-Faster, Smarter, Better.

[3] Canterburg RJ, Clavet CJ, Mc Garvey EL, Koopman C (1998). HIV risk-related attitudes and behaviours of incarcerated adolescents: implications for public school students. High School Journal..

[4] Hall PA, Holmqvist M, Sherry SB (2004). Risky adolescent sexual behaviour: A psychological perspective for primary care clinicians. http://www.medscape.com/viewarticle/467059-5.

[5] Irwin CE, Igra V, Eyre S, Millstein S (2005). Risk-taking behaviour in adolescents: the paradigm. http://www.stanford.edu/~kcobb/femstudies/LectureOne.htm.

[6] Gilborn LZ (2002). The effect of HIV infection and AIDS on children in Africa. West J Med..

[7] Cote AM, Sobela F, Dzokoto A, Nzambi K, Asamoah-Adu C, Labbe AC, Masse B, Mensah J, Frost E, Pepin J (2004). Transactional sex is the driving force in the dynamics of HIV in Accra, Ghana. AIDS..

[8] Hargreaves JP, Bonell CP, Morison LA, Kim JC, Phetla G, Porter JD, Watts C, Pronyk PM (2007). Explaining continued high HIV prevalence in South Africa: socioeconomic factots, HIV incidence and sexual behaviour change among rural cohort, 2001-2004. AIDS..

[9] Nyindo M (2005). Complementary factors contributing to the rapid spread of HIV-1 in Sub-Saharan Africa: a review. East Afr Med J..

[10] Volk JE, Prestage G, Jin F, kaldor J, Ellard J, Kippax S, Grulich AE (2006). Risk factor for HIV seroconversion in homosexual men in Australia. Sex Health..

[11] Agardh A, Emmelin M, Muriisa R, Ostergren PO (2010). Social capital and sexual behavior among Uganda University students. Glob Health Action.

[12] Agardh A, Cantor-Graae E, Ostergren PO (2012). Sexual Risk-Taking Behavior, and Mental Health: a study of University Students in Uganda. Int J Behav Med..

[13] Bless C, Higson-Smith C (2000). Fundamentals of social research methods: an African perspective.

[14] Rwenge M (2000). Sexual risk behaviours among young people in Bamenda, Cameroon. International Family Planning Perspectives..

[15] Ntaganira J, Hass LJ, Hosner S, Brown L, Mock NB (2012). Sexual risk behaviors among youth heads of household in Gikongoro, South Province of Rwanda. BMC Public Health..

[16] Haddison EC, Ngeufack-Tsague G, Noubom M, Mbatcham W, Ndumbe PM, Mbopi-Keou X (2012). Voluntary counseling and testing for HIV among high school students in the Tiko Health District, Cameroon. Pan Afr Med J..

[17] Dingeta T, Oljira L, Assefa N (2012). Patterns of sexual risk behavior among undergraduate university students in Ethiopia: a cross-sectional study. Pan Afr Med J..

[18] Rwenge MJR (2003). Poverty and sexual risk behaviour among young people in Bamenda, Cameroon. African Population Studies..

[19] Gueye M, Castle S, Konate M (2001). Timing of first intercourse among Malian adolescents: implications for contraceptive use. International Family Planning Perspectives..

[20] Makhetha TE (1996). Adolescent pregnancy and birth outcomes among adolescents aged 13 to 16 in Soweto. Unpublished Master's dissertation.

[21] Murray NJ, Zabin LS, Toledo-Dreves V, Lvengo Charath X (1998). Gender differences in the factors influencing first intercourse among urban students in Chile. International Family Planning Perspectives..

[22] Zaleski EH, Schiaffino KM (2000). Religiosity and sexual risk-taking behaviour during the transition to college. J Adolescence..

[23] Moore JP, Burton DR (1999). HIV-1 neutralising antibiotics: how full is the bottle?. Nature Medicines..

[24] Mouton J (2001). How to succeed in your Master's and Doctoral studies. A South African guide and resource book.

